# Invasive lizard has fewer parasites than native congener

**DOI:** 10.1007/s00436-021-07233-5

**Published:** 2021-07-07

**Authors:** Beatriz Tomé, D. James Harris, Ana Perera, Isabel Damas-Moreira

**Affiliations:** 1 CIBIO-InBIO, FCUP, University of Porto, Porto, Portugal; 2 Department of Biology, University of Porto, Porto, Portugal; 3 Department of Biological Sciences, Macquarie University, Sydney, Australia; 4 Department of Behavioural Ecology, Bielefeld University, Bielefeld, Germany

**Keywords:** Biological invasions, Reptile, *Podarcis siculus*, *Podarcis virescens*, Haemogregarines

## Abstract

Invasive species can carry parasites to introduced locations, which may be key to understand the success or failure of species establishment and the invasive potential of introduced species. We compared the prevalence and infection levels of haemogregarine blood parasites between two sympatric congeneric species in Lisbon, Portugal: the invasive Italian wall lizard (*Podarcis siculus*) and the native green Iberian wall lizard (*Podarcis virescens*). The two species had significant differences in their infection levels: while *P. virescens* had high prevalence of infection (69.0%), only one individual of *P. siculus* was infected (3.7%), and while *P. virescens* exhibited an average intensity of 1.36%, the infected *P. siculus* individual had an infection rate of only 0.04%. Genetic analyses of 18S rRNA identified two different haemogregarine haplotypes in *P. virescens*. Due to the low levels of infection, we were not able to amplify parasite DNA from the infected *P. siculus* individual, although it was morphologically similar to those found in *P. virescens*. Since other studies also reported low levels of parasites in *P. siculus*, we hypothesize that this general lack of parasites could be one of the factors contributing to its competitive advantage over native lizard species and introduction success.

## Introduction

Biological invasions are a contemporary global problem, with major economic and ecological impacts, and are directly linked to the current loss of biodiversity (Simberloff et al. 2013). Although research regarding biological invasions has expanded extensively in recent decades, less attention has been given to the role of parasites in this phenomenon. However, the interchange of parasites is of major concern, whether the transmission is from introduced to native species (spillover) or vice versa (spillback) (Wells et al. 2015). Parasites can have notable impact on host communities, ultimately shaping the biodiversity distribution and the structure of ecosystems (Tompkins et al. 2011). As such, parasites may be crucial to understand the success or failure of species establishment and the invasive potential of introduced species, through either their presence or absence (Poulin 2017). Indeed, the “enemy release hypothesis” predicts that as an introduced species is no longer exposed to the predators and pathogens from their native range, it will have a competitive advantage over native species in the new colonized habitats (Colautti et al. 2004). On the other hand, if an introduced host species carries parasites, and settles quickly and efficiently in the new location, the native host species might be exposed to these new parasites and will have no time to adapt to this threat. This is of particular concern when introduced and native host species are closely related and ecologically similar, as this increases the probability and speed of parasite interchange, since for example their immunology should be similar (Young et al. 2017).

In the current study, we compare the infection by haemogregarines (blood parasites from the phylum Apicomplexa) between two congeneric lizard species that live in sympatry in Lisbon, Portugal: the invasive Italian wall lizard (*Podarcis siculus*) and the native green Iberian wall lizard (*Podarcis virescens*). *Podarcis siculus* is native to the Italian Peninsula and the Adriatic coast, but has numerous introduced populations worldwide, having arrived to Lisbon 20 years ago (González de la Vega et al. 2001). This lizard represents a risk to native lizards as it can outcompete, hybridize, or displace them (e.g. Capula et al. 2002; Damas-Moreira et al. 2020). *Podarcis virescens* is a native species of the Iberian Peninsula, which occupies central Spain and southern Portugal (Geniez et al. 2014) and has no reported introductions elsewhere. These two lizard species have overlapping habitat and presumably similar dietary preferences (Arnold and Burton 2002; Ribeiro and Sá-Sousa 2018), and therefore compete for similar resources. In Lisbon, the two lizards occur in sympatry, and present differences in their behaviour and competition skills (Ribeiro and Sá-Sousa 2018; Damas-Moreira et al. 2019, 2020). Given the recent origin of introduction of *P. siculus*, these two species, one a known invasive species and the other showing no evidence for this, can be useful to study the interchange of parasites between closely related native and introduced host species, and its role in establishment success.

## Materials and methods

In the spring of 2017, 27 adult males of *P. siculus* and 29 adult males of *P. virescens* were collected from the urban gardens in Parque das Nações, Lisbon, Portugal (N 38° 45′ 43, W 9° 5′ 41). Females were not included in the study, as their reproductive status might affect infection levels (Maia et al. 2014). Blood was collected on slides for microscopy and on Whatman paper for genetic characterization. Prevalence (the percentage of infected individuals in a population) was determined by screening the blood slides for haemogregarines at 400 × magnification under an Olympus CX41 microscope with an in-built digital camera (SC30; Olympus, Hamburg, Germany). To estimate the intensity (percentage of infected cells per 2500 erythrocytes), five random areas of each slide were photographed at 400 × magnification with the cell^B software (Olympus, Münster, Germany), and 500 erythrocytes per area were counted in ImageJ v.1.50b (as in, e.g., Maia et al. 2014). Statistical differences in prevalence between lizard species were estimated using Fisher’s exact test performed in R v.3.3.1 (R Core Team 2016). No statistical analysis could be performed on the parasite intensity values as only one *P. siculus* individual was infected.

For the genetic characterization, we extracted DNA from the blood of all infected individuals using standard high-salt methods, while PCR reactions were performed using primers designed to amplify a 600 bp long region of the 18S rRNA gene of haemogregarines, HepF300 and HepR900, following Maia et al. (2012). The amplified products were purified and sequenced by an external company (Genewiz, UK). Sequences were compared to the GenBank database using a BLAST search to confirm the identity of the amplified products. We then conducted phylogenetic analyses (Bayesian Inference and Maximum Likelihood) using the same methodology as in Tomé et al. (2021). The final alignment matrix was 579 base pairs long and included 205 partial 18S rRNA sequences from haemogregarines. Two of these were new sequences from this study, which are available in the GenBank database under the accession numbers MZ327715 and MZ327716.

## Results and discussion

Our study shows that the invasive *P. siculus* was less parasitized than the native *P. virescens*, both in terms of prevalence (p-value < 0.001) and intensity of infection. In fact, only one individual of the invasive species was infected (out of 27, 3.7%) while we found 20 infected individuals of the native species (out of 29, 69.0%). This single *P. siculus* individual presented a very low intensity (0.04%, representing only 1 parasite per 2500 erythrocytes). The mean intensity among infected individuals of the native species was 1.36% ± 2.14 SD (i.e. an average of 34 parasites per 2500 erythrocytes), and intensity values ranged from 0.04 to 9.88%. The prevalence and intensity levels here detected are congruent with results from previous studies. They are usually high in *P. virescens*, although the values can vary significantly across populations (e.g. Maia et al. 2014). Regarding *P. siculus*, available data in its native range (near Rome, Italy) shows very low intensity levels (average lower than 0.1%; Sacchi et al. 2011), while in populations introduced to the USA no blood parasites were detected (Burke et al. 2007).

**Fig. 1:**
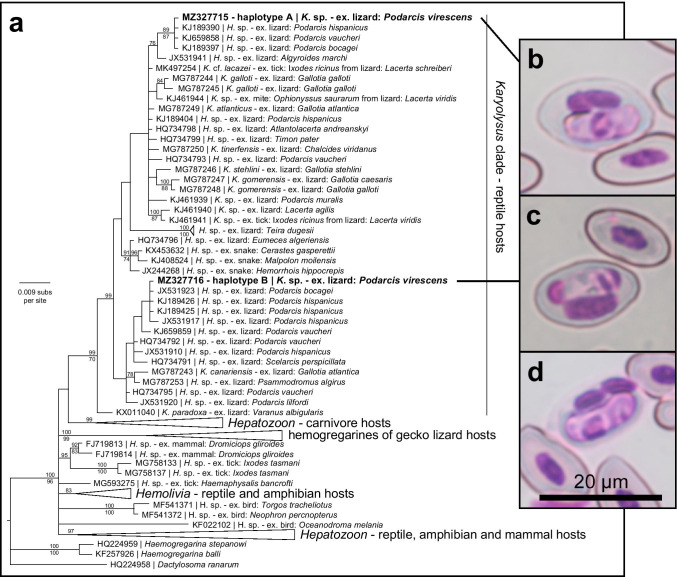
Phylogenetic tree and photographs of gametocytes of the haemogregarines found in this study. (a) Tree derived from the Bayesian Inference analysis of haemogregarine 18S rRNA gene sequences. Bayesian posterior probabilities are given above relevant nodes and below are the bootstrap values from the Maximum Likelihood analysis (only values over 70 are shown). New sequences from this study are identified in bold and larger font size. For simplicity, some branches of the phylogenetic tree have been collapsed. (b) Gametocytes found in *P. virescens* of haplotype A and (c) haplotype B. (d) Gametocyte from the only *P. siculus* infected individual

All the amplified parasites were genetically identified as haemogregarines (within a clade considered the genus *Karyolysus*), the most common blood parasite in reptiles (Telford 2009). We detected two different haemogregarine haplotypes that differed by 2.6% (15 nucleotides out of 572 bp alignment) infecting the native lizard species (referred here as A and B, see Fig. 1a for the phylogenetic tree). These were identical to haplotypes infecting other *Podarcis* species of the Iberian Peninsula and Morocco, including *P. hispanicus* and *P. bocagei*. Due to the low intensity, it was not possible to retrieve sequences for all infections, including the single *P. siculus* individual infected. However, morphologically it resembled haemogregarines bearing haplotype A (photographs of gametocytes infecting erythrocytes available in Fig. 1b for haplotype A, Fig. 1c for haplotype B, and Fig. 1d for the infection in *P. siculus*). Because there are no comparable genetic sequences available for haemogregarines infecting *P. siculus*, from either native or introduced populations, it was not possible to determine the identity and closest relatives of the haemogregarines infecting *P. siculus* and confirm if there was any haemogregarine interchange between the two host species. Nevertheless, several haemogregarine haplotypes that infect lizards in the Iberian Peninsula are shared across lacertid species (Harris et al. 2012; Maia et al. 2012, 2014), which suggest that these parasites are not species specific, and so they may potentially infect *P. siculus*.

Despite living in the same urban habitat and sharing similar resources, *P. virescens* and *P. siculus* clearly differed in parasite infection levels. Unfortunately, with the current information, we cannot assess whether the haemogregarines infecting *P. siculus* are parasites brought from the source populations, or whether they have been transferred by the sympatric *P. virescens*. The introduced *P. siculus* population in Lisbon has its origin in Tuscany, Italy (Silva-Rocha et al. 2012), where interestingly it also exhibited low parasite levels (Sacchi et al. 2011). Potentially, there could be some mechanism limiting haemogregarine spread from the native to the introduced lizard species. Such barriers may include spatial segregation between the two host species, immunological differences, or transmission vector affinity towards a specific host species (Poulin 2017). It is unlikely that spatial segregation promotes differences in parasite infection given it would occur across a micro-scale and that the two host species can punctually overlap in Lisbon (Ribeiro and Sá-Sousa 2018). On the other hand, immunological differences between the two species might be supported by the low parasite intensity in *P. siculus*, both in Lisbon and in Tuscany. Likewise, vectors may also help explain our results. Because haemogregarines need a multiple host lifecycle, it can complicate parasite establishment, as a suitable vector (in this case ticks or mites) also needs to be present in the introduced location (Poulin 2017). Nevertheless, as we found one parasite in one *P. siculus* individual, and because different host lacertid species can share parasite haplotypes (Harris et al. 2012; Maia et al. 2012, 2014), there should be little obstacle for transmission to *P. siculus*.

Another aspect that needs further investigation is the effect of the haemogregarines on lizard hosts. Haemogregarine infections can cause changes in basking behaviour, metabolism, and reproductive effort, and even anaemia and mortality (see Telford 2009). Conversely, studies on flight escape distance (an indicator of antipredator behaviour) found no correlation with haemogregarine infection loads (e.g. Damas-Moreira et al. 2014). Overall, the general picture of low infections by parasites in *P. siculus* and the possible negative effects of haemogregarines suggest enemy release might indeed provide this lizard with some competitive advantage over infected native populations. Or alternatively, infection in *P. virescens* might give the native species a competition handicap that enabled *P. siculus* to establish and persist at least locally. For example, in another congener lizard pair on the Caribbean island of St. Maarten, *Anolis gingivinus* and *Anolis wattsi* only co-occur in locations where the former is heavily parasitized by *Plasmodium azurophilum*, while throughout the island *A. gingivinus* outcompetes *A. wattsi* (which is rarely infected). This pattern is observed even within distances of only a few hundred meters. The malaria parasite was suggested to mediate competition between the two lizards, allowing the competitively inferior lizard to coexist (Schall, 1992), although a later study (Perkins, 2001) found the two species of anoles still cohabited despite no *P. azurophilum* infections being identified. This suggests that either the parasite was not playing a role, or that its effect on competition plays out over a longer timeframe.

Furthermore, several other traits can explain *P. siculus*’s colonization success, including versatile diet and habitat choice, and adaptable behaviour and morphology (e.g. Vervust et al. 2007; Damas-Moreira et al. 2019, 2020). Future studies should focus on identifying the haemogregarines infecting *P. siculus* in its native and introduced ranges, on understanding the effect of haemogregarines on host fitness, and on monitoring the long-term fluctuations of parasite infections in these and other similar species pairs. Although the particular role of haemogregarines in the introduction success of *P. siculus* is still not clear, host-parasite dynamics can undoubtedly be a crucial factor in both the success of an introduction and the impact on native communities.

## Data Availability

Dataset is available at OSF: https://doi.org/10.17605/OSF.IO/C4WTP.
